# Side-Specific Prognostic Factors in Colon Cancer: A Retrospective Analysis of Right- and Left-Sided Tumors

**DOI:** 10.3390/cancers17203315

**Published:** 2025-10-14

**Authors:** Michał Serafin, Magdalena Mąka, Julia Szostek, Iga Kania, Beata Jabłońska, Sławomir Mrowiec

**Affiliations:** 1 Student Scientific Society, Department of Digestive Tract Surgery, Faculty of Medical Sciences in Katowice, Medical University of Silesia, 14 Medyków Street, 40-752 Katowice, Poland; michal.j.serafin@gmail.com (M.S.); s81151@365.sum.edu.pl (M.M.); s81394@365.sum.edu.pl (J.S.); s80989@365.sum.edu.pl (I.K.); 2 Department of Digestive Tract Surgery, Faculty of Medical Sciences in Katowice, Medical University of Silesia, 14 Medyków Street, 40-752 Katowice, Poland; smrowiec@sum.edu.pl

**Keywords:** colon cancer, left-sided colon cancer, right-sided colon cancer, predictive factors, overall survival

## Abstract

Background: Tumor sidedness in colon cancer has been linked to biological and clinical differences, but its impact on survival and prognostic factors remains unclear. This study aimed to find the predictors of overall survival (OS) in patients with right-sided colon cancer (RCC) and left-sided colon cancer (LCC) undergoing surgical treatment. Methods: A retrospective single-center study was conducted on 247 patients with colon cancer, including 117 with RCC and 130 with LCC. Clinical, surgical, and pathological variables were analyzed. Cox regression and ROC curve analyses were used to identify independent predictors of OS in the overall cohort and tumor-side subgroups. Results: RCC patients were older (69 vs. 68 years, *p* = 0.03), had lower hemoglobin levels (11.7 vs. 12.95 g/dL, *p* < 0.01), and more often presented with anemia (34.18% vs. 11.48%, *p* < 0.001). LCC patients more frequently underwent emergency surgery (13.74% vs. 5.69%, *p* = 0.03). Mucinous adenocarcinomas were more frequent in RCC (12.82% vs. 5.38%, *p* = 0.03), whereas distant metastases (15.38% vs. 6.84%, *p* = 0.03) and liver metastases (14.61% vs. 6.84%, *p* = 0.04) were more common in LCC. The one-year overall survival was similar between LCC and RCC before (88.09% vs. 91.52%, *p* = 0.15) and after propensity score matching (89.32% vs. 91.87%, *p* = 0.60) In multivariate Cox regression, independent predictors of lower OS included advanced AJCC stage (HR = 34.54, *p* < 0.001) in RCC, while, in LCC, AJCC stage (HR = 31.14, *p* = 0.001 and stoma (HR = 5.86, *p* = 0.01) were significant. Tumor location itself was not associated with OS (*p* = 0.18). Conclusions: Prognostic factors in colon cancer vary with tumor location. Side-specific risk stratification may improve outcome prediction and guide personalized management.

## Simple Summary

Colon cancer remains a significant global health challenge, with growing evidence that tumors originating on the right and left sides of the colon represent distinct disease entities. These differences include variations in patient characteristics, tumor biology, and survival outcomes. In this retrospective analysis of 247 patients, we compared clinicopathological features and prognostic factors between right- and left-sided tumors. Our findings demonstrate that tumor location is associated with measurable differences in prognosis, suggesting that side-specific factors should be considered when planning treatment strategies. This knowledge may contribute to more precise risk stratification and individualized management approaches in clinical practice.

## 1. Introduction

Colorectal cancer (CRC) ranks among the top three most common malignancies worldwide, with over 1.9 million new cases and 935,000 deaths estimated in 2020, and its incidence is projected to rise to 2.5 million new cases annually by 2035 [1,2]. While CRC is primarily a disease that affects older populations, increasing incidence among younger adults has brought renewed focus to its biological and clinical heterogeneity [3]. A growing body of evidence indicates that right-sided colon cancer (RCC) and left-sided colon cancer (LCC) represent distinct disease entities rather than simply different anatomical locations. These differences arise from their embryological origin, vascular supply, molecular profiles, and metastatic patterns. Specifically, RCC develops from the embryonic midgut and is supplied by the superior mesenteric artery, whereas LCC originates from the hindgut and receives blood from the inferior mesenteric artery [4,5,6]. Furthermore, RCC is more frequently associated with mucinous histology and peritoneal spread, while LCC more often presents with liver and lung metastases [5,6]. Such fundamental differences underscore the importance of analyzing colon cancer outcomes in a side-specific manner.

Molecularly, RCC is more often associated with mucinous histology, microsatellite instability, and BRAF or RAS mutations, whereas LCC tends to exhibit better differentiation and more favorable oncologic profiles [7,8]. While surgical resection remains the standard curative approach for colon cancer, the impact of tumor sidedness on prognosis remains controversial. Some studies report worse outcomes for RCC [6,9,10], while others show no significant differences between RCC and LCC [11,12,13,14].

Previous research has identified a number of clinicopathological and systemic factors as predictors of outcome in CRC. For example, histological subtype plays an important role, with mucinous adenocarcinoma being consistently associated with poorer prognosis compared to conventional adenocarcinoma [15,16]. In addition, systemic inflammation has emerged as a strong determinant of survival. Elevated C-reactive protein (CRP) levels and derived indices, such as the CRP-to-albumin ratio (CAR), have been repeatedly linked to worse overall survival in colorectal cancer [17,18,19,20]. Similarly, hematological markers such as the neutrophil-to-lymphocyte ratio (NLR) and carcinoembryonic antigen (CEA) have been validated as prognostic indicators in both resectable and advanced CRC [21,22]. However, the majority of these analyses have considered colorectal cancer as a single entity, potentially overlooking prognostic differences attributable to tumor sidedness.

This study aimed to determine clinical and pathological factors associated with early postoperative complications and overall survival in patients operated on for right- and left-sided colon cancer. The analysis was based on a single-center cohort and focused on evaluating prognostic differences between RCC and LCC to support more tailored treatment approaches.

## 2. Materials and Methods

### 2.1. Design of the Study

The retrospective analysis of electronic and paper medical records of all patients treated for CRC at the Department of Digestive Tract Surgery, Medical University of Silesia in Katowice, Poland, between January 2014 and December 2023 was performed.

Study inclusion criteria included primary surgical colon resection in elective or emergency mode and cancer confirmed by histopathological finding; exclusion criteria were rectal cancer, recurrent colon cancer, and lack of postoperative follow-up.

The study included 247 adult patients (135; 54.65% males and 112; 45.34% females) with a mean age of 69 (37–92), IQR 12 years.

### 2.2. Inclusion Criteria to Surgical Treatment

All patients were assessed by a multidisciplinary team comprising surgeons, radiologists, and oncologists, who collaboratively determined eligibility for a specific treatment modality based on computed tomography (CT) findings. Additionally, endoscopic biopsy followed by histopathological confirmation of colon cancer was performed in 231 patients (92.03%) prior to surgical intervention.

The cohort was stratified into two subgroups based on tumor localization: RCC (117; 47.36%), including tumors located in the cecum, ascending colon, and the proximal two-thirds of the transverse colon; and LCC (130; 52.63%), encompassing tumors in the distal third of the transverse colon, descending colon, and sigmoid colon.

The OS was defined as the duration of a patient’s life from the date of the surgical procedure to either the date of death or the date of the last recorded contact.

### 2.3. Analyzed Data

Collected data included demographics, comorbidities, clinical symptoms, blood parameters (including CRP, albumin, neutrophil/lymphocyte ratio (NLR), and CRP/albumin ratio (CAR)), ASA classification, operative details (type, duration, emergency vs. elective), margin status, histopathological findings (tumor size, grade, lymphovascular and perineural invasion), lymph node involvement, distant metastasis, and follow-up outcomes. The primary endpoint was overall survival (OS), defined as the time from surgery to death or last contact.

Complications, reoperations, mortality, and hospital stay were recorded. Tumor histopathology was obtained from surgical and pathology reports; follow-up data were retrieved from clinic records.

Nutritional Risk Screening 2002 (NRS 2002) was assessed according to European Society for Clinical Nutrition and Metabolism recommendations. The score combines evaluation of recent weight loss, body mass index, reduced dietary intake, and disease severity, with an additional point assigned to patients aged ≥70 years. The total score ranges from 0 to 7 points. Patients with an NRS ≥ 3 were considered to be at risk of malnutrition [23].

Weight loss was defined as an unintentional loss of more than 5% of body weight within the 6 months preceding diagnosis or hospital admission [24].

Anemia was defined as hemoglobin < 13 g/dL in men and <11.5 g/dL in women [25].

### 2.4. Statistical Analysis

Statistical analyses were performed using Statistica^®^ version 13.3 (StatSoft, Tulsa, OK, USA). Categorical variables were reported as counts and percentages, and continuous variables as means with standard deviation (SD), medians with interquartile range (IQR), as appropriate. Normality was assessed using the Shapiro–Wilk test. Group comparisons between RCC and LCC were made using the chi-square test, Fisher’s exact test, or the Mann–Whitney U test.

Univariate logistic regression was used to identify predictors of postoperative complications, followed by multivariate logistic regression with significant variables. OS was evaluated using Kaplan–Meier analysis and Cox proportional hazards regression. Variables significant in univariate Cox models were entered into multivariate analysis. Receiver operating characteristic (ROC) curves were generated for significant continuous predictors of OS from the final Cox models. While we report 1-year overall survival for descriptive purposes, the Cox regression analysis included the full follow-up period available for each patient, with censoring applied accordingly. Therefore, predictors identified in the Cox model refer to overall survival (OS) and are not limited to the 1-year mark. A *p*-value < 0.05 was considered statistically significant.

## 3. Results

### 3.1. Preoperative Patients’ Data

The RCC patients’ group was older compared to the LCC group (69 vs. 68, *p* = 0.04). In patients with RCC, anemia was more often observed compared to the patients with LCC (34.18% vs. 11.48%, *p* < 0.001). Additionally, more patients in the LCC group reported hematochezia compared to the RCC group (22.30% vs. 12.82%); however, this result was close to statistical significance (*p* = 0.06).

Patients with RCC had lower median hemoglobin level compared to the LCC patients (11.7 vs. 12.95, *p* < 0.001) (Table 1).

### 3.2. Procedure Characteristics and Early Outcome

More patients in the LCC group were admitted in emergency mode compared to the RCC group (13.84% vs. 4.27%, *p* = 0.01).

More patients in the RCC group underwent colectomy with primary anastomosis compared to the LCC group (92.31% vs. 74.61%, *p* = 0.001). In addition, in the whole cohort, six (2.43%) patients underwent simultaneous synchronous liver metastases resection. Five (1.97%) patients underwent anatomical liver resection, four (1.57%) segmental resection of the 4th liver segment, and one (0.40%) resection of 7th liver segment, while one (0.40%) patient underwent non-anatomical resection of the right liver lobe.

Thirty-nine (17.40%) complications were observed. Thirty-day mortality was 2.02% (Table 2).

### 3.3. Predictive Factors for Postoperative Complications

In the univariate logistic regression analysis for postoperative complications, in the whole cohort, the CRP (*p* < 0.001), CAR (*p* = 0.005), emergency admission mode (*p* < 0.001), laparoscopic surgery (*p* = 0.04), primary colon anastomosis (*p* < 0.001), and >400 mL blood loss (*p* = 0.03) proved to influence the occurrence of postoperative complications. On the other hand, in subgroup analysis in RCC, the presence of comorbidities (*p* = 0.04) and CRP (*p* = 0.02) influenced postoperative complications, while, in LCC, the older age (*p* = 0.04), albumin levels (*p* = 0.03), CRP (*p* = 0.006), CAR (*p* = 0.01), emergency admission mode (*p* < 0.001), and primary colon anastomosis (*p* = 0.005) were significant factors.

The univariate logistic regression analyses of the whole cohort, RCC, and LCC are shown in Table 3.

Multivariate logistic regression analysis showed no statistically significant predictors of postoperative complications in the overall cohort and in the RCC and LCC subgroups.

### 3.4. Histopathological Data of the Tumor

The most common tumor localization in patients with LCC was sigmoid colon (99; 76.05%), while, in the RCC group, it was ascending colon (47; 40.17%).

More mucinous adenocarcinomas were observed in the RCC group compared to the LCC group (12.82% vs. 5.38%, *p* = 0.045). Additionally, more G3 tumors were observed in patients with RCC than in patients with LCC (10.26% vs. 2.31%, *p* = 0.03). There were no statistically significant differences between groups in terms of pathological T (*p* = 0.86) and N (*p* = 0.43) staging; however, more distant metastases were observed in the LCC group compared to the RCC group (15.38% vs. 6.84%, *p* = 0.04). Although a higher proportion of LCC patients were diagnosed at stage IV compared to RCC, this difference did not reach statistical significance when tested across the entire AJCC distribution (*p* = 0.12). Liver metastases were observed more often in patients with LCC compared to the patients with RCC (14.61% vs. 6.84%, *p* = 0.04). This finding is potentially associated with an observed higher overall rate of distant metastases in the LCC group. RCC was associated with a significantly higher lymph node isolation than LCC (18 vs. 13; *p* < 0.001). This difference likely reflects the greater extent of lymphadenectomy inherent to right hemicolectomy—the predominant operation for RCC in our study—compared with sigmoidectomy in LCC (Table 4).

### 3.5. Long-Term Outcome After Surgical Treatment of Colon Cancer

Median follow-up time was 16, IQR 30.5 months.

In the follow-up period, we observed seven (5.69%) and 14 (10.94%) deaths in the RCC and LCC subgroups, respectively. All deaths were related to cancer-related complications

**Figure 1:**
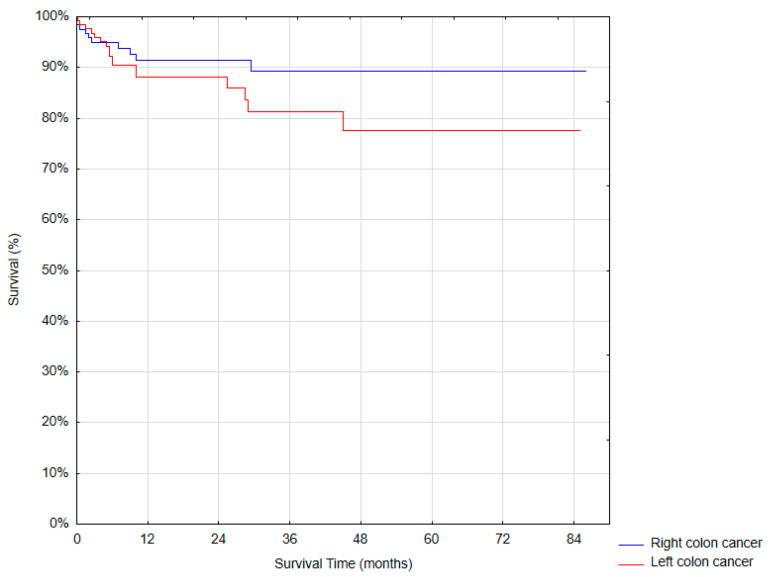
Survival rates of the series in right vs. left colon cancer (Log-rank test *p* = 0.18).

One-year overall survival after the surgical treatment was 89.76% (Table 5, Figure 1).

### 3.6. Predictor Factor for 1-Year Survival After Surgical Treatment of Colon Cancer

The univariate Cox proportional hazard regression model analyses in the whole cohort, RCC, and LCC are shown in Table 6.

In multivariate analysis with the Cox proportional hazard regression model, in the whole cohort, complications (HR = 10.65, *p* = 0.001), AJCC stage (III–IV, HR = 21.59, *p* < 0.001), and stoma (HR = 5.48, *p* = 0.01) were independent predictive factors for 1-year-survival.

Among patients with RCC in multivariate analysis, the AJCC stage (III–IV, HR = 34.54, *p* < 0.001) proved to be an independent predictive factor for 1-year-survival.

In the LCC group in multivariate analysis, stoma (HR = 5.86, *p* = 0.01) and AJCC stage (III–IV, HR = 31.14, *p* = 0.001) were independent predictive factors for 1-year-survival.

### 3.7. Propensity Score Matching

To directly compare the influence of sidedness of colon cancer (RCC vs. LCC), propensity score matching (PSM) was performed with variables found to be statistically different in earlier analysis (occurrence of distant metastasis, admission mode, and primary anastomosis as matched variables). Due to the clinically insignificant difference in age between RCC and LCC (median difference of 1 year), this variable was excluded from the analysis. Due to problems with PSM, hemoglobin level and occurrence of anemia were also excluded from the analysis.

The matched cohort can be found in Table 7.

## 4. Discussion

In our cohort of 247 colon cancer patients, several independent predictors of OS were identified. In multivariate analysis, postoperative complications, stoma formation, and advanced AJCC stage were significantly associated with poorer 1-year survival. Subgroup analysis revealed location-specific patterns: in LCC, stoma and AJCC stage were significant, while, in RCC, only AJCC stage remained as an independent predictor. Tumor sidedness itself was not associated with 1-year survival before (*p* = 0.18) and after propensity score matching (*p* = 0.60).

Several studies have reported worse OS in RCC compared to LCC, including Hodges et al., who found a 5-year OS of 60.85% in RCC versus 65.2% in LCC, with better outcomes in LCC across all Duke’s stages [4]. However, others, such as Yang et al., found no significant difference (HR = 1.23, *p* = 0.11) [26]. Similarly, our analysis showed no OS difference between RCC and LCC at 1 year before (*p* = 0.18) and after PSM (*p* = 0.60). While stage distribution has been proposed as a key explanation for survival differences between RCC and LCC, other contributing factors must also be considered. Molecular characteristics such as MSI status, BRAF and KRAS mutations, and mucinous histology are more frequent in RCC and have been consistently associated with adverse prognosis [7,8]. Treatment-related factors may also contribute, as anti-EGFR therapies are typically reserved for left-sided, RAS wild-type tumors, whereas right-sided tumors may derive less benefit from such regimens. Moreover, surgical strategies such as complete mesocolic excision are more often applied in right-sided resections, potentially affecting long-term outcomes [12,13,14]. In our study, the lack of molecular and treatment-related data, such as RAS/BRAF status or the type and timing of systemic therapy, may have limited our ability to capture some of the side-specific prognostic differences described in previous reports.

In our whole cohort, the occurrence of postoperative complications was associated with lower 1-year survival; however, this was not reflected in subgroup analysis of RCC and LCC. Similar results can be found in the Warps et. al. study, where patients without postoperative complications had 73.2% 5-year OS, reduced to 51.8% in patients with occurrence of complications. Moreover, postoperative complications were associated with an almost 1.6 times higher risk of death [27]. The literature indicates that postoperative complications negatively influence survival, though the underlying mechanisms vary [28,29,30]. Surgical complications may promote tumor progression through inflammatory responses [31,32,33], whereas medical complications often reflect pre-existing comorbidities and can impair overall fitness [34,35,36,37]. In addition, several studies report that patients who experience complications are less likely to receive timely adjuvant chemotherapy, further worsening their prognoses [35,36]. The lack of impact of postoperative complications in subgroup analysis in our study is likely related to the limited sample size and the number of events in each subgroup, which reduced statistical power to detect such an effect.

Stoma formation was an independent predictor of reduced 1-year survival in the overall cohort (HR = 5.48, *p* = 0.01), as well as in LCC (HR = 5.86, *p* = 0.01) subgroup. Similar results were reported by Asghari-Jafarabadi et al. [37]. This association may be explained by the high rate of stoma-related complications, reported in up to 70% of patients [38], and its frequent use in emergency or palliative settings—both associated with advanced disease, worse baseline status, and delays in adjuvant therapy.

Advanced AJCC stage (III–IV) was a strong predictor of reduced 1-year survival in the overall cohort (HR = 21.56, *p* < 0.001) and in RCC (HR = 34.54, *p* < 0.001) and LCC (HR = 31.14, *p* = 0.001), consistent with findings from Mangone and Bustamante-Lopez et al. [39,40].

Mucinous histology was not associated with reduced 1-year survival in the overall cohort or in RCC and LCC. The literature on this topic remains inconsistent. While a large population-based study by Warschkow et al. [41] found no OS difference between mucinous and non-mucinous tumors, several clinical studies [42,43,44,45] reported worse prognosis and lower chemotherapy response in mucinous adenocarcinomas. These differences may stem from biological features such as higher rates of microsatellite instability, KRAS/BRAF mutations, and mucin overexpression (MUC2, MUC5AC), all linked to aggressive behavior and treatment resistance [46,47]. In our study, insignificance may reflect the small number of mucinous cases.

This study has several limitations, including its single-center design and moderate sample size, which may limit generalizability and reduce statistical power, particularly in subgroup analyses. Furthermore, part of the study period overlapped with the COVID-19 pandemic, potentially affecting timely access to diagnostics and surgery, thus contributing to more advanced disease at presentation. Moreover, due to the retrospective design of our study, we were not able to analyze molecular markers of RCC and LCC, which might have influenced the predictive results Despite these limitations, the study provides a comprehensive side-specific evaluation of prognostic factors in colon cancer. Larger, multicenter prospective studies are necessary to validate these results and to further explore the prognostic relevance of tumor sidedness.

## 5. Conclusions

This study identified several clinical and pathological factors associated with overall survival in patients undergoing surgery for colon cancer. Independent predictors of poorer outcomes included advanced tumor stage, stoma formation, and occurrence of postoperative complications. These associations varied by tumor location, with advanced tumor stage and stoma formation being particularly relevant in left-sided tumors, while advanced tumor stage had greater prognostic value in right-sided cancers. Tumor sidedness alone did not influence survival, highlighting the importance of individualized risk assessment beyond anatomical location.

## Figures and Tables

**Table 1:** Preoperative patients’ data.

Variable	RCC (*n* = 117; 47.36%)	LCC (*n* = 130; 52.63%)	Total (*n* = 247)	*p*
Age (years)	69 (43–92), IQR 12	68 (37–87), IQR 13	69 (37–92), IQR 11	0.03
Gender				
Male	63 (53.84%)	72 (55.38%)	135 (54.65%)	0.78
Female	53 (46.15%)	58 (44.62%)	112 (45.34%)	
BMI (kg/m^2^)	26.13 (17.47–47.11), IQR 5.33	26.94 (17.63–41.66), IQR 5.90	26.72 (17.47–47.11), IQR 5.60	0.63
Presence of comorbidities (yes)	93 (75.61%)	91 (69.47%)	184 (72.44%)	0.27
Clinical symptoms (yes)	70 (59.82%)	80 (61.53%)	150 (60.72%)	0.80
Abdominal pain	39 (33.33%)	44 (33.85%)	83 (33.60%)	1
Weight loss	25 (21.37%)	30 (23.07%)	55 (22.26%)	0.88
Anemia	40 (34.18%)	15 (11.48%)	55 (22.26%)	<0.001
Hematochezia	15 (12.82%)	29 (22.30%)	44 (17.81%)	0.06
Constipation	12 (10.27%)	17 (13.07%)	29 (11.75%)	0.55
Nausea and vomiting	15 (12.82%)	10 (7.69%)	25 (10.12%)	0.17
Body weakness	15 (12.82%)	10 (7.69%)	25 (10.12%)	0.20
Diarrhea	5 (4.27%)	10 (7.69%)	15 (6.07%)	0.30
Melaena	3 (2.56%)	2 (1.54%)	5 (2.02%)	0.68
Colon obstruction	1 (0.85%)	3 (2.3%)	4 (1.62%)	0.60
Colon perforation	2 (1.60%)	0 (0%)	2 (0.80%)	0.47
Preoperative blood tests				
Albumin (g/dL)	4.31 (2.2–5.0), IQR 0.51	4.38 (2.90–5.26), IQR 0.66	4.33 (2.2–5.26) IQR 0.6	0.41
White blood cells (G/L)	7.07 (3.61–27.5), IQR 3.18	7.3 (3.33–26.54), IQR 4	7.14 (3.33–27.5), IQR 3.49	0.62
Neutrophiles (G/L)	4.53 (1.18–91.6), IQR 2.57	4.78 (0.35–74.90), IQR 3.56	4.61 (0.35–91.6), IQR 3.17	0.88
Lymphocytes (G/L)	1.54 (0.47–20.10), IQR 0.77	1.42 (0.34–22.40), IQR 0.92	1.46 (0.34–22.4), IQR 0.83	0.42
Hemoglobin (g/dL)	11.7 (6.2–18), IQR 4.3	12.95 (8–18.1), IQR 3	12.6 (6.2–18.1), IQR 3.40	<0.001
Platelets (G/L)	291 (73–689), IQR 124	273 (92–775), IQR 113	282 (73–775), IQR 120	0.12
CRP (mg/L)	7.67 (0.6–262.05), IQR 33.61	8.45 (0.4–278.2), IQR 41.25	7.87 (0.4–278.2), IQR 34.50	0.49
CAR	1.89 (0.13–65.90), IQR 8.46	2.21 (0.12–55.39), IQR 11.79	2.01 (0.12–65.89), IQR 9.88	0.24
NLR	3.05 (0.22–114.5), IQR 2.30	3.37 (0.26–43.40), IQR 2.61	3.19 (0.22–114.5), IQR 2.41	0.62
NRS score	2 (2–6), IQR 1	2 (2–6), IQR 1	2 (2–6) IQR 1	0.93
Preoperative endoscopic biopsy (yes)	113 (93.45%)	118 (90.85%)	231 (92.03%)	0.44

Abbreviations: RCC—right colon cancer, LCC—left colon cancer, CAR—C-reactive protein level to albumin level ratio, NRL—neutrophiles to lymphocytes ratio, NRS—nutritional risk score, SD—standard deviation, IQR—interquartile range.

**Table 2:** Procedural characteristics and early outcome.

Variable	RCC (*n* = 117; 47.36%)	LCC (*n* = 130; 52.63%)	Total (*n* = 247)	*p*
Admission mode				
Elective mode	112 (95.72%)	112 (86.15%)	224 (90.67%)	0.01
Emergency mode	5 (4.27%)	18 (13.84%)	23 (9.31%)	
ASA score				
I	1 (0.85%)	2 (1.54%)	3 (1.21%)	0.72
II	53 (45.44%)	56 (43.07%)	108 (44.72%)	
III	57 (48.71%)	61 (46.92%)	118 (47.77%)	
IV	7 (5.98%)	10 (7.6+%)	17 (6.88%)	
V	0 (0%)	1 (0.78%)	1 (0.4%)	
Scope of surgery				
Radical tumor resection	112 (95.73%)	115 (88.46%)	227 (91.90%)	0.06
Palliative resection	5 (4.27%)	15 (11.53%)	20 (8.09%)	
Type of surgical access				
Laparotomy	78 (66.66%)	84 (64.61%)	162 (65.57%)	0.79
Laparoscopy	39 (33.33%)	46 (35.38%)	85 (34.41%)	
Type of surgery				
Right hemicolectomy	101 (86.32%)	0 (0%)	101 (40.89%)	
Sigmoidectomy	0 (0%)	90 (69.23%)	90 (36.44%)	
Left hemicolectomy	0 (0%)	25 (19.23%)	25 (10.12%%)	
Right extended hemicolectomy	15 (12.82%)	0 (0%)	15 (6.07%)	
Left extended hemicolectomy	0 (0%)	8 (6.15%)	8 (3.24%)	
Transverse colon resection	1 (0.85%)	7 (5.38%)	8 (3.24%)	
Primary anastomosis (yes)	108 (92.31%)	97 (74.61%)	205 (84.00%)	0.001
Type of anastomosis				
Side to side ileo-transversostomy	63 (51.12%)	0 (0%)	63 (24.80%)	
End to end descendo-rectostomy	0 (0%)	60 (45.80%)	60 (23.62%)	
End to end transverso-sigmoidostomy	0 (0%)	22 (16.79%)	22 (8.66%)	
End to end ileo-transversostomy	21 (17.07%)	0 (0%)	21 (8.27%)	
Side to end ileo-transversostomy	16 (13.01%)	0 (0%)	16 (6.30%)	
End to side ileo-transversostomy	8 (7.32%)	0 (0%)	8 (3.54%)	
End to end descendo-sigmoidostomy	0 (0%)	4 (3.05%)	4 (1.57%)	
Side to side descendo-sigmoidostomy	0 (0%)	3 (2.31%)	3 (1.18%)	
End to end ascendo-descendostomy	0 (0%)	2 (1.62%)	2 (0.79%)	
Side to side transverso-sigmoidostomy	0 (0%)	2 (1.62%)	2 (0.79%)	
End to end sigmoido-sigmoidostomy	0 (0%)	2 (1.62%)	2 (0.79%)	
Side to side ileo-sigmoidostomy	0 (0%)	1 (0.77%)	1 (0.40%)	
End to side ileo-descendostomy	0 (0%)	1 (0.77%)	1 (0.40%)	
Primary stoma (yes)	9 (7.69%)	33 (25.38%)	42 (17.00%)	0.001
Type of stoma (construction type)				
End stoma	8 (7.32%)	29 (22.31%)	37 (14.98%)	0.002
Loop stoma	1 (0.85%)	4 (3.05%)	5 (2.02%)	
Type of stoma (time type)				
Definitive stoma	8 (7.32%)	24 (18.32%)	37 (14.57%)	0.001
Reversal stoma	1 (0.85%)	9 (6.92%)	10 (4.00%)	
Stoma localization				
Descending colostomy	0 (0%)	27 (20.61%)	27 (10.93%)	
Ileostomy	7 (5.98%)	0 (0%)	7 (2.83%)	
Transverse colostomy	0 (0%)	6 (4.62%)	6 (2.43%)	
Ascending colostomy	2 (1.71%)	0 (0%)	2 (0.79%)	
Treatment of liver metastasis (yes)	3 (2.44%)	3 (2.29%)	6 (2.43%)	1
Type of surgical treatment of liver metastasis				
Anatomical liver resection	2 (1.71%)	3 (2.31%)	5 (1.97%)	
Non-anatomical liver resection	1 (0.85%)	0 (0%)	1 (0.40%)	
Blood loss				
<400 mL	113 (95.83%)	123 (94.62%)	235 (95.14%)	0.42
>400 mL	5 (4.27%)	7 (5.38%)	12 (4.85%)	
Duration of the procedure (minutes)	215 (80–420), IQR 85	205 (100–505), IQR 85	210 (80–505), IQR 90	0.45
Complications	18 (15.38%)	21 (16.15%)	39 (17.40%)	0.50
Wound infection	5 (4.27%)	4 (3.07%)	9 (3.64%)	0.74
Wound dehiscence	3 (2.56%)	5 (3.84%)	8 (3.23%)	0.72
Colon obstruction	1 (0.85%)	3 (2.31%)	4 (1.61%)	1
Descending colostomy obstruction	0 (0%)	3 (2.31%)	3 (1.21%)	
Ileo-transversostomy obstruction	1 (0.85%)	0 (0%)	1 (0.40%)	
Intestine perforation	0 (0%)	3 (2.31%)	3 (1.21%)	0.24
Descending colon perforation	0 (0%)	2 (1.62%)	2 (0.81%)	
Ileum perforation	0 (0%)	1 (0.77%)	1 (0.40%)	
Anastomotic leakage	4 (3.41%)	0 (0%)	4 (1.61%)	0.05
Ileo-transverse anastomosis	4 (3.41%)	0 (0%)	4 (1.61%)	
Sepsis	2 (1.63%)	1 (0.77%)	3 (1.21%)	0.54
Intra-abdominal abscess	1 (0.85%)	1 (0.77%)	2 (0.81%)	1
Colo-cutaneus fistula	1 (0.85%)	1 (0.77%)	2 (0.81%)	1
Intra-abdominal hemorrhage	1 (0.85%)	0 (0%)	1 (0.40%)	0.48
Cutaneo-vesical fistula	0 (0%)	1 (0.77%)	1 (0.40%)	1
Clostridium difficile infection	1 (0.85%)	0 (0%)	1 (0.40%)	0.48
Iatrogenic ureteral perforation	0 (0%)	1 (0.77%)	1 (0.40%)	1
Acute ischemia of ileum	0 (0%)	1 (0.77%)	1 (0.40%)	1
Reoperations	3 (2.56%)	6 (4.62%)	9 (3.64%)	0.71
30-day mortality	2 (1.71%)	3 (2.31%)	5 (2.02%)	1
Duration of hospitalization (days)	8 (1–39), IQR 4	8 (1–33), IQR 5	8 (1–39), IQR 4.5	0.88
Rehospitalization	3 (2.56%)	4 (3.07%)	7 (2.83%)	1

Abbreviations: ASA—American Society of Anesthesiologist, RCC—right colon cancer, LCC—left colon cancer, SD—standard deviation, IQR—interquartile range.

**Table 3:** Univariate logistic regression analysis of predictive factor for postoperative complications after surgical treatment of colon cancer.

	Univariate Analysis (Overall)			Univariate Analysis (RCC)			Univariate Analysis (LCC)		
Variable	OR	95% CI	*p*	OR	95% CI	*p*	OR	95% CI	*p*
Age	1.02	0.99–1.1	0.16	1	0.99–1.05	0.82	1.05	1.01–1.1	0.04
Gender									
Male	1			1		0.39	1		0.33
Female	1.05	0.54–1.71	0.86	0.64	0.23–1.75		1.55	0.25–1.55	
BMI	1.01	0.95–1.09	0.59	1	0.92–1.06	0.61	1.01	0.91–1.11	0.82
Presence of comorbidities	0.76	0.37–1.69	0.45	0.33	0.11–0.95	0.04	1.51	0.55–4.08	0.41
Albumin (g/dL)	0.47	0.22–1.22	0.05	0.62	0.31–3.01	0.45	0.31	0.13–0.93	0.03
White blood cells (G/L)	1.06	0.97–1.15	0.16	1.08	0.94–1.21	0.22	1.06	0.93–1.17	0.41
Neutrophiles (G/L)	0.97	0.92–1.04	0.45	0.96	0.82–1.12	0.63	0.98	0.92–1.05	0.61
Lymphocytes (G/L)	0.88	0.59–1.39	0.57	1	0.72–1.38	0.99	0.6+	0.25–1.59	0.37
Hemoglobin (g/dL)	0.96	0.83–1.14	0.58	0.93	0.76–1.22	0.54	0.95	0.78–1.17	0.62
Platelets (G/L)	1	0.99–1	0.18	1	0.99–1	0.17	1	0.99–1	0.66
CRP (mg/L)	1.01	1.01–1.02	<0.001	1.00	1–1.02	0.02	1.01	1.01–1.02	0.006
CAR	1.04	1.01–1.07	0.005	1.03	0.98–1.07	0.19	1.06	1.01–1.2	0.01
NLR	1	0.96–1.04	0.89	0.99	0.89–1.07	0.74	1.01	0.95–1.08	0.77
NRS score	1.24	0.85–1.88	0.29	1.38	0.76–2.5	0.29	1.13	0.65–1.9	0.64
ASA score									
I–II	1		0.07	1		0.21	1		0.20
III–V	2.07	0.93–4.24		1.89	0.7–5.06		1.97	0.68–6.18	
Admission mode									
Emergency mode	5.5	2.24–13.52	<0.001	4	0.61–19.81	0.14	6	2.09–17.40	<0.001
Elective mode	1			1			1		
Surgical approach									
Laparotomy	1		0.04	1		0.39	1		0.08
Laparoscopy	0.44	0.20–0.97		0.52	0.15–2.06		0.39	0.14–1.14	
Scope of surgical treatment									
Radical tumor resection	1		0.13	1		0.15	1		
Palliative resection	2.20	0.79–5.74		4	0.61–22.21		1.65	0.48–5.67	0.44
Primary anastomosis	0.32	0.15–0.61	<0.001	0.6	0.11–1.04	0.55	0.27	0.10–0.71	0.005
Stoma	1			1			1		
Blood loss									
>400 mL	3.70	1.11–9.79	0.03	4	0.61–22.21	0.15	3.44	0.71–12.39	0.12
<400 mL	1			1			1		
Duration of procedure	1	0.99–1	0.95	0.99	0.99–1	0.19	1	0.9–1.01	0.36

Abbreviations: ASA—American Society of Anesthesiologist, BMI—body mass index, CAR—C-reactive protein to albumin ratio, CRP—C-reactive protein, LCC—left colon cancer, NRL—neutrophil to lymphocyte ratio, NRS—nutritional risk score, OR—odds ratio, RCC—right colon cancer, 95% CI—95% confidence interval.

**Table 4:** Histopathological tumor data.

Variable	RCC (*n* = 117; 47.36%)	LCC (*n* = 130; 52.63%)	Total (*n* = 247)	*p*
Tumor localization				
Sigmoid colon	0 (0%)	99 (76.15%)	99 (40.08%)	
Ascending colon	47 (40.17%)	0 (0%)	47 (19.02%)	
Caecum	31 (26.49%)	0 (0%)	31 (12.55%)	
Hepatic flexure	23 (19.69%)	0 (0%)	23 (9.31%)	
Proximal ⅔ of transverse colon	15 (12.82%)	0 (0%)	15 (6.07%)	
Splenic flexure	0 (0%)	15 (11.54%)	15 (6.07%)	
Descending colon	0 (0%)	10 (7.69%)	10 (4.04%)	
Distal ⅓ of transverse colon	0 (0%)	6 (4.61%)	6 (2.42%)	
Ileocecal valve	1 (0.81%)	0 (0%)	1 (0.40%)	
Tumor size (mm)	40 (2–220), IQR 35	35 (4–450), IQR 25	40 (2–450), IQR 25	0.09
Resection margin status				
R0	117 (100%)	128 (98.46%)	245 (99.10%)	0.17
R2	0 (0%)	2 (1.54%)	2 (0.80%)	
Histological type				
Adenocarcinoma	102 (87.18%)	123 (94.61%)	225 (91.09%)	0.045
Mucinous tumor	15 (12.82%)	7 (5.38%)	22 (8.91%)	
Grading				
G1	18 (15.38%)	24 (18.46%)	42 (17%)	0.03
G2	87 (74.36%)	103 (79.23%)	190 (76.92%)	
G3	12 (10.26%)	3 (2.31%)	15 (6.07%)	
Pathological staging				
T				
0	3 (2.56%)	1 (0.77%)	4 (1.61%)	0.86
1	16 (13.68%)	17 (13.07%)	33 (13.36%)	
2	25 (21.37%)	27 (20.76%)	52 (21.05%)	
3	54 (46.15%)	65 (50.00%)	119 (48.17%)	
4	19 (16.24%)	20 (15.38%)	39 (15.78%)	
N				
0	76 (62.39%)	86 (66.14%)	159 (64.37%)	0.43
1	28 (23.93%)	33 (25.38%)	61 (24.69%)	
2	16 (13.67%)	11 (8.46%)	27 (10.93%)	
M				
0	109 (93.16%)	110 (84.61%)	226 (88.66%)	0.04
1	8 (6.84%)	20 (15.38%)	28 (11.36%)	
AJCC stage				
I	38 (32.47%)	40 (30.77%)	78 (31.57%)	0.12
II	34 (29.05%)	40 (30.76%)	74 (29.95%)	
III	37 (31.62%)	30 (23.07%)	67 (27.12%)	
IV	8 (6.84%)	20 (15.27%)	28 (11.33%)	
Localization of distant metastasis *				
Liver	8 (6.84%)	19 (14.61%)	27 (10.93%)	0.04
Lung	1 (0.85%)	0 (0%)	1 (0.40%)	0.48
Peritoneum	0 (0%)	1 (0.77%)	1 (0.40%)	1
Lymph nodes isolated	18 (1–45), IQR 10	13 (3–57), IQR 9	16 (1–57), IQR 10	<0.001
Lymphovascular invasion (yes)	23 (19.65%)	23 (17.69%)	46 (18.62%)	0.81
Perineural invasion (yes)	6 (5.12%)	5 (3.84%)	11 (4.45%)	0.68

* Footnote; One patient with RCC had lungs and liver metastasis simultaneously. Abbreviations: RCC—right colon cancer, LCC—left colon cancer, SD—standard deviation, IQR—interquartile range, AJCC—American Joint Committee on Cancer.

**Table 5:** Long-term outcome of patients treated for colon cancer.

Variable	RCC (*n* = 117; 47.36%)	LCC (*n* = 130; 52.63%)	Total (*n* = 247)	*p*
Follow-up duration (months)	18.5 (0.3–86), IQR 34.5	13.5 (0.3–85), IQR 24.5	16 (0.3–86), IQR 30.5	0.14
Chemotherapy (yes)	19 (16.23%)	35 (26.92%)	52 (21.05%)	0.05
FOLFIRI	4 (3.42%)	12 (9.23%)	16 (6.48%)	0.07
FOLFOX4	2 (1.71%)	12 (9.23%)	14 (5.67%)	0.05
XELOX	7 (5.98%)	6 (4.58%)	13 (5.12%)	0.77
LF4	3 (2.56%)	2 (1.54%)	5 (2.02%)	0.67
mFOLFOX4	1 (0.85%)	1 (0.77%)	1 (0.40%)	1
LF1	0 (0%)	1 (0.77%)	1 (0.40%)	1
Carboplatin	1 (0.85%)	0 (0%)	1 (0.40%)	0.48
mFOLFOX6	0 (0%)	1 (0.77%)	1 (0.40%)	1
De Gramont	1 (0.85%)	0 (0%)	1 (0.40%)	0.48
Radiotherapy (yes)	0 (0%)	3 (2.31%)	3 (1.21%)	0.24
Recurrence (yes)	0 (0%)	2 (1.55%)	2 (0.80%)	0.49
1-year overall survival	91.52% (SE 2.74%)	88.09% (SE 3.14%)	89.76% (SE 2.10%)	0.15
Estimated 5-year overall survival	89.29% (SE 3.47%)	77.58% (SE 5.81%)	83.53% (SE 3.38%)	0.15

Abbreviations: RCC—right colon cancer, LCC—left colon cancer, SD—standard deviation, IQR—interquartile range, SE—standard error.

**Table 6:** Univariate Cox proportional hazard regression model analysis on 1-year survival rates.

	Univariate Analysis (Overall)			Univariate Analysis (RCC)			Univariate Analysis (LCC)		
Variable	HR	95% CI	*p*	HR	95% CI	*p*	HR	95% CI	*p*
Age	1.02	0.97–1.06	0.56	1.06	0.97–1.13	0.18	1	0.95–1.05	0.93
Gender									
Male	1		0.58	1		0.58	1		0.23
Female	1.24	0.58–2.70		0.69	0.18–2.54		1.79	0.65–4.84	
BMI	1.03	0.94–1.05	0.50	0.96	0.87–1.06	0.42	1.09	0.98–1.13	0.1
Presence of comorbidities	1.14	0.48–2.81	0.76	1.18	0.24–5.71	0.83	1.20	0.42–3.36	0.72
Clinical symptoms	3.2	1.20–8.45	0.02	2.65	0.60–13.41	0.22	3.63	1.04–12.67	0.04
Albumin (g/dL)	0.82	0.34–1.94	0.65	0.63	0.16–2.47	0.51	0.74	0.23–2.42	0.62
White blood cells (G/L)	1.12	1.05–1.20	<0.001	1.08	0.95–1.22	0.19	1.21	1.09–1.35	0.001
Neutrophiles (G/L)	1	0.96–1.04	0.90	0.61	0.19–1.98	0.41	1.02	0.98–1.06	0.37
Lymphocytes (G/L)	0.96	0.68–1.35	0.80	0.88	0.24–3.38	0.85	0.99	0.74–1.34	0.97
Hemoglobin (g/dL)	0.93	0.79–1.11	0.47	0.72	0.53–0.98	0.04	1.01	0.84–1.36	0.57
Platelets (G/L)	1.01	1.008–1.014	0.03	1.01	1–1.01	0.23	1.07	1.007–1.01	0.04
CRP (mg/L)	1	0.99–1	0.73	1	0.99–1.01	0.92	1	0.99–1.01	0.72
CAR	1.02	0.98–1.05	0.39	1	0.92–1.08	0.98	1.04	0.99–1.08	0.11
NLR	1	0.96–1.04	0.93	0.69	0.23–2.05	0.51	1.02	0.96–1.10	0.45
NRS score	1.46	0.99–2.17	0.06	1.51	0.84–2.71	0.18	1.55	0.88–2.75	0.13
ASA score									
I–II	1		0.04	1		0.2	1		0.11
III–V	2.42	1.05–5.53		2.43	0.62–9.43		2.33	0.82–6.64	
Admission mode									
Emergency mode	3.53	1.41–8.81	<0.001	4.21	1.21–27.63	0.18	2.89	1.02–8.35	0.04
Elective mode	1			1			1		
Primary anastomosis	1		<0.001	1		0.03	1		<0.001
Stoma	6.77	3.12–14.63		4.82	1.22–19.12		7.55	2.75–20.71	
Complications	3.34	1.53–7.31	0.003	6.32	1.82–21.99	0.004	2.19	0.77–6.29	0.14
Tumor type									
Adenocarcinoma	1			1					
Mucinous tumor	1.29	1.01–1.65	0.04	1.38	0.97–1.98	0.07	3.72	0.85–16.37	0.08
Tumor size (mm)	1	0.99–1	0.57	1.01	0.99–1.03	0.06	1	0.99–1.01	0.91
AJCC stage									
0–II	1			1			1		
III–IV	21.10	4.9–89.35	<0.001	28.29	2.14–133.42	<0.001	12.50	2.85–54.01	<0.001
Number of isolated lymph nodes									
<12	1			1 *			1		
12–21	1.71	0.66–4.42	0.26				1.02	0.36–2.95	0.96
>21	1.19	0.38–3.69	0.76				1.57	0.39–6.30	0.52
Lymphovascular invasion	1.43	0.58–3.56	0.43	0.61	0.07–4.55	0.54	1.82	0.64–5.16	0.26
Postoperative chemotherapy	2.01	0.93–4.33	0.07	3.22	0.91–11.42	0.06	1.37	0.52–3.63	0.55
Pre-pandemic years (2014–2019)	1			1			1		
Pandemic years (2020–2023)	0.29	0.12–72	0.006	0.22	0.04–1.05	0.06	0.35	0.12–1.01	0.05

* Footnote: Because fewer than five deaths were reported in one of the analyzed groups, it was not possible to perform the Cox proportional hazard regression analysis. Abbreviations: ASA—American Society of Anesthesiologist, AJCC—American Joint Committee on Cancer, BMI—body mass index, CAR—C-reactive protein to albumin ratio, CRP—C-reactive protein, LCC—left colon cancer, NRL—neutrophil to lymphocyte ratio, NRS—nutritional risk score, HR—hazard ratio, RCC—right colon cancer, 95% CI—95% confidence interval.

**Table 7:** Comparison of RCC and LCC after propensity score matching.

Variable	RCC (*n* = 117; 49.57%)	LCC (*n* = 115; 50.43%)	*p*
Distant metastases	8 (6.84%)	15 (13.04%)	0.13
Admission mode			
Emergency	5 (4.27%)	6 (5.22%)	0.77
Elective	112 (95.73%)	109 (94.78%)	
Primary anastomosis	108 (92.31%)	104 (90.44%)	0.65
RCC vs. LCC survival comparison			
Follow-up time	18.5 (0.3–86)	14 (0.5–85)	0.31
30-day mortality	2 (1.71%)	2 (1.74%)	1
12-month survival	91.87% SE 2.79%	89.32% SE 3.22%	0.60
Estimated 5-year survival	89.51% SE 3.58%	86.62% SE 4.11%	0.60
Cox proportional hazard regression model			
	HR	95% CI	*p*
RCC	1		
LCC	1.25	0.52–3.03	0.60

Abbreviations: LCC—left colon cancer, HR—hazard ratio, RCC—right colon cancer, 95% CI—95% confidence interval, SE—standard error.

## Data Availability

Data supporting the results are available from the corresponding authors on request.

## References

[1] Global Cancer Statistics 2020: GLOBOCAN Estimates of Incidence and Mortality Worldwide for 36 Cancers in 185 Countries-Sung-2021-CA: A Cancer Journal for Clinicians-Wiley Online Library. https://acsjournals.onlinelibrary.wiley.com/doi/10.3322/caac.21660.

[2] Colorectal Cancer-ScienceDirect. https://www.sciencedirect.com/science/article/pii/S0140673619323190?via%3Dihub.

[3] Benelkhaiat R. (2017). Colorectal Cancer in Marrakech Tensift Elhaouz, Epidemiological and Anatomopathological Aspects, About 2584 Cases in 19 Years. Int. J. Adv. Res..

[4] Hodges N., Mackenzie H., D’Souza N., Brown G., Miskovic D. (2022). Survival Outcomes for Right-versus Left-Sided Colon Cancer and Rectal Cancer in England: A Propensity-Score Matched Population-Based Cohort Study. Eur. J. Surg. Oncol. J. Eur. Soc. Surg. Oncol. Br. Assoc. Surg. Oncol..

[5] Hugen N., van de Velde C.J.H., de Wilt J.H.W., Nagtegaal I.D. (2014). Metastatic Pattern in Colorectal Cancer Is Strongly Influenced by Histological Subtype. Ann. Oncol. Off. J. Eur. Soc. Med. Oncol..

[6] Benedix F., Kube R., Meyer F., Schmidt U., Gastinger I., Lippert H., the Colon/Rectum Carcinomas (Primary Tumor) Study Group (2010). Comparison of 17,641 Patients With Right- and Left-Sided Colon Cancer: Differences in Epidemiology, Perioperative Course, Histology, and Survival. Dis. Colon Rectum.

[7] Missiaglia E., Jacobs B., D’Ario G., Di Narzo A., Soneson C., Budinska E., Popovici V., Vecchione L., Gerster S., Yan P. (2014). Distal and proximal colon cancers differ in terms of molecular, pathological, and clinical features. Ann. Oncol..

[8] Petrelli F., Tomasello G., Borgonovo K., Ghidini M., Turati L., Dallera P., Passalacqua R., Sgroi G., Barni S. (2017). Prognostic Survival Associated With Left-Sided vs Right-Sided Colon Cancer: A Systematic Review and Meta-Analysis. JAMA Oncol..

[9] Holch J.W., Ricard I., Stintzing S., Modest D.P., Heinemann V. (2017). The Relevance of Primary Tumour Location in Patients with Metastatic Colorectal Cancer: A Meta-Analysis of First-Line Clinical Trials. Eur. J. Cancer Oxf. Engl. 1990.

[10] West N.P., Morris E.J.A., Rotimi O., Cairns A., Finan P.J., Quirke P. (2008). Pathology Grading of Colon Cancer Surgical Resection and Its Association with Survival: A Retrospective Observational Study. Lancet Oncol..

[11] Weiss J.M., Pfau P.R., O’Connor E.S., King J., LoConte N., Kennedy G., Smith M.A. (2011). Mortality by Stage for Right- versus Left-Sided Colon Cancer: Analysis of Surveillance, Epidemiology, and End Results—Medicare Data. J. Clin. Oncol. Off. J. Am. Soc. Clin. Oncol..

[12] Bernhoff R., Sjövall A., Buchli C., Granath F., Holm T., Martling A. (2018). Complete Mesocolic Excision in Right-Sided Colon Cancer Does Not Increase Severe Short-Term Postoperative Adverse Events. Colorectal Dis. Off. J. Assoc. Coloproctology G. B. Irel..

[13] Lee L., Erkan A., Alhassan N., Kelly J.J., Nassif G.J., Albert M.R., Rt Monson J. (2018). Lower Survival after Right-Sided versus Left-Sided Colon Cancers: Is an Extended Lymphadenectomy the Answer?. Surg. Oncol..

[14] Bertelsen C.A., Neuenschwander A.U., Jansen J.E., Tenma J.R., Wilhelmsen M., Kirkegaard-Klitbo A., Iversen E.R., Bols B., Ingeholm P., Rasmussen L.A. (2019). 5-Year Outcome after Complete Mesocolic Excision for Right-Sided Colon Cancer: A Population-Based Cohort Study. Lancet Oncol..

[15] Kanemitsu Y., Kato T., Hirai T., Yasui K., Morimoto T., Shimizu Y., Kodera Y., Yamamura Y. (2003). Survival after Curative Resection for Mucinous Adenocarcinoma of the Colorectum. Dis. Colon Rectum.

[16] Arfa N., Hamdani I., Gharbi L., Ben Abid S., Ghariani B., Mannai S., Mestiri H., Khalfallah M.T., Mzabi S.R. (2006). Survival and prognostic factors of colorectal adenocarcinoma: Analytic multifactor review of 150 cases. Ann. Chir..

[17] Deme D., Telekes A. (2017). Prognostic importance of plasma C-reactive protein (CRP) in oncology. Orv. Hetil..

[18] Li C., Xu Q., Chen L., Luo C., Ying J., Liu J. (2017). C-Reactive Protein (CRP) as a Prognostic Factor for Colorectal Cancer after Surgical Resection of Pulmonary Metastases. Bull. Cancer.

[19] Shibutani M., Maeda K., Nagahara H., Iseki Y., Ikeya T., Hirakawa K. (2016). Prognostic Significance of the Preoperative Ratio of C-Reactive Protein to Albumin in Patients with Colorectal Cancer. Anticancer Res..

[20] Zhou Q.P., Li X.J. (2019). C-Reactive Protein to Albumin Ratio in Colorectal Cancer: A Meta-Analysis of Prognostic Value. Dose-Response.

[21] Tsai P.-L., Su W.-J., Leung W.-H., Lai C.-T., Liu C.-K. (2016). Neutrophil-Lymphocyte Ratio and CEA Level as Prognostic and Predictive Factors in Colorectal Cancer: A Systematic Review and Meta-Analysis. J. Cancer Res. Ther..

[22] Mazaki J., Katsumata K., Kasahara K., Tago T., Wada T., Kuwabara H., Enomoto M., Ishizaki T., Nagakawa Y., Tsuchida A. (2020). Neutrophil-to-lymphocyte ratio is a prognostic factor for colon cancer: A propensity score analysis. BMC Cancer.

[23] Hersberger L., Bargetzi L., Bargetzi A., Tribolet P., Fehr R., Baechli V., Geiser M., Deiss M., Gomes F., Kutz A. (2020). Nutritional risk screening (NRS 2002) is a strong and modifiable predictor risk score for short-term and long-term clinical outcomes: Secondary analysis of a prospective randomised trial. Clin. Nutr..

[24] Hernández J.L., Riancho J.A., Matorras P., González-Macías J. (2003). Clinical evaluation for cancer in patients with involuntary weight loss without specific symptoms. Am. J. Med..

[25] World Health Organization (2024). Guideline on Haemoglobin Cutoffs to Define Anaemia in Individuals and Populations.

[26] Yang C.-Y., Yen M.-H., Kiu K.-T., Chen Y.-T., Chang T.-C. (2022). Outcomes of Right-Sided and Left-Sided Colon Cancer after Curative Resection. Sci. Rep..

[27] Warps A.K., Tollenaar R.A.E.M., Tanis P.J., Dekker J.W.T., Dutch ColoRectal Audit (2022). Postoperative complications after colorectal cancer surgery and the association with long-term survival. Eur. J. Surg. Oncol..

[28] van der Sijp M.P., Bastiaannet E., Mesker W.E., van der Geest L.G., Breugom A.J., Steup W.H., Marinelli A.W., Tseng L.N., Tollenaar R.A., van de Velde C.J. (2016). Differences between colon and rectal cancer in complications, short-term survival and recurrences. Int. J. Colorectal Dis..

[29] Breugom A.J., van Dongen D.T., Bastiaannet E., Dekker F.W., van der Geest L.G., Liefers G.J., Marinelli A.W., Mesker W.E., Portielje J.E., Steup W.H. (2016). Association Between the Most Frequent Complications After Surgery for Stage I-III Colon Cancer and Short-Term Survival, Long-Term Survival, and Recurrences. Ann. Surg. Oncol..

[30] Klaver C.E.L., Wasmann K.A.T.G.M., Verstegen M., van der Bilt J.D.W., Nagtegaal I.D., van Ramshorst B., Tanis P.J., Wolthuis A.M., van Santvoort H.C., de Wilt J.H.W. (2018). Postoperative abdominal infections after resection of T4 colon cancer increase the risk of intra-abdominal recurrence. Eur. J. Surg. Oncol..

[31] Desborough J.P. (2000). The stress response to trauma and surgery. Br. J. Anaesth..

[32] Coussens L.M., Werb Z. (2002). Inflammation and cancer. Nature.

[33] Rettig T.C., Verwijmeren L., Dijkstra I.M., Boerma D., van de Garde E.M., Noordzij P.G. (2016). Postoperative Interleukin-6 Level and Early Detection of Complications After Elective Major Abdominal Surgery. Ann. Surg..

[34] Merkow R.P., Bentrem D.J., Mulcahy M.F., Chung J.W., Abbott D.E., Kmiecik T.E., Stewart A.K., Winchester D.P., Ko C.Y., Bilimoria K.Y. (2013). Effect of postoperative complications on adjuvant chemotherapy use for stage III colon cancer. Ann. Surg..

[35] Des Guetz G., Nicolas P., Perret G.Y., Morere J.F., Uzzan B. (2010). Does delaying adjuvant chemotherapy after curative surgery for colorectal cancer impair survival? A meta-analysis. Eur. J. Cancer..

[36] Arhi C.S., Burns E.M., Bouras G., Aylin P., Ziprin P., Darzi A. (2019). Complications after discharge and delays in adjuvant chemotherapy following colonic resection: A cohort study of linked primary and secondary care data. Colorectal Dis..

[37] Asghari-Jafarabadi M., Wilkins S., Plazzer J.P., Yap R., McMurrick P.J. (2024). Prognostic Factors and Survival Disparities in Right-Sided versus Left-Sided Colon Cancer. Sci. Rep..

[38] Shabbir J., Britton D.C. (2010). Stoma Complications: A Literature Overview. Colorectal Dis. Off. J. Assoc. Coloproctol. G B Irel..

[39] Mangone L., Pinto C., Mancuso P., Ottone M., Bisceglia I., Chiaranda G., Michiara M., Vicentini M., Carrozzi G., Ferretti S. (2021). Colon Cancer Survival Differs from Right Side to Left Side and Lymph Node Harvest Number Matter. BMC Public Health.

[40] Bustamante-Lopez L.A., Nahas S.C., Nahas C.S.R., Pinto R.A., Marques C.F.S., Cecconello I. (2019). IS THERE A DIFFERENCE BETWEEN RIGHT- VERSUS LEFT-SIDED COLON CANCERS? DOES SIDE MAKE ANY DIFFERENCE IN LONG-TERM FOLLOW-UP?. ABCD Arq. Bras. Cir. Dig. São Paulo.

[41] Warschkow R., Tarantino I., Huttner F.J., Schmied B.M., Guller U., Diener M.K., Ulrich A. (2016). Predictive Value of Mucinous Histology in Colon Cancer: A Population-Based, Propensity Score Matched Analysis. Br. J. Cancer.

[42] Luo C., Cen S., Ding G., Wu W. (2019). Mucinous Colorectal Adenocarcinoma: Clinical Pathology and Treatment Options. Cancer Commun..

[43] Negri F.V., Wotherspoon A., Cunningham D., Norman A.R., Chong G., Ross P.J. (2005). Mucinous Histology Predicts for Reduced Fluorouracil Responsiveness and Survival in Advanced Colorectal Cancer. Ann. Oncol..

[44] Catalano V., Loupakis F., Graziano F., Torresi U., Bisonni R., Mari D., Fornaro L., Baldelli A.M., Giordani P., Rossi D. (2009). Mucinous Histology Predicts for Poor Response Rate and Overall Survival of Patients with Colorectal Cancer and Treated with First-Line Oxaliplatin- and/or Irinotecan-Based Chemotherapy. Br. J. Cancer.

[45] Mekenkamp L.J.M., Heesterbeek K.J., Koopman M., Tol J., Teerenstra S., Venderbosch S., Punt C.J.A., Nagtegaal I.D. (2012). Mucinous Adenocarcinomas: Poor Prognosis in Metastatic Colorectal Cancer. Eur. J. Cancer.

[46] Maisano R., Azzarello D., Maisano M., Mafodda A., Bottari M., Egitto G., Nardi M. (2012). Mucinous Histology of Colon Cancer Predicts Poor Outcomes with FOLFOX Regimen in Metastatic Colon Cancer. J. Chemother..

[47] Basile D., Rosati G., Bergamo F., Garattini S.K., Banzi M., Zampino M., Bozzarelli S., Marchetti P., Galli F., Galli F. (2023). Prognostic Value of Body Mass Index in Stage II/III Colon Cancer: Posthoc Analysis From the TOSCA Trial. Clin. Colorectal Cancer.

